# Identifying predictive factors for mortality in patients with TBI at a neurosurgery department

**DOI:** 10.25122/jml-2023-0114

**Published:** 2023-04

**Authors:** Iulia-Sevastiana Pastor, Ioana Para, Ștefan Cristian Vesa, Ioan Ștefan Florian

**Affiliations:** 1 Department of Neurosurgery, Faculty of Medicine, Iuliu Hațieganu University of Medicine and Pharmacy, Cluj-Napoca, Romania; 2 4^th^ Department of Internal Medicine, Faculty of Medicine, Iuliu Hațieganu University of Medicine and Pharmacy, Cluj-Napoca, Romania; 3 Pharmacology, Toxicology and Clinical Pharmacology, Faculty of Medicine, Iuliu Hațieganu University of Medicine and Pharmacy, Cluj-Napoca, Romania

**Keywords:** traumatic brain injury, predictive factors, days of hospitalization

## Abstract

Traumatic brain injury (TBI) can have severe consequences in most cases. Many therapeutic and neurosurgical strategies have been improved to optimize patient outcomes. However, despite adequate surgery and intensive care, death can still occur during hospitalization. TBI often results in protracted hospital stays in neurosurgery departments, indicating the severity of brain injury. Several factors related to TBI are predictive of longer hospital stays and in-hospital mortality rates. This study aimed to identify predictive factors for intrahospital days of death due to TBI. This was a longitudinal, retrospective, analytical, observational study that included 70 TBI-related deaths admitted to the Neurosurgery Clinic in Cluj-Napoca for a period of four years (January 2017 to December 2021) using a cohort model. We identified some clinical data related to intrahospital death after TBI. The severity of TBI was classified as mild (n=9), moderate(n=13), and severe (n=48) and was associated with significantly fewer hospital days (p=0.009). Patients with associated trauma, such as vertebro-medullary or thoracic trauma, were more likely to die after a few days of hospitalization (p=0.007). Surgery applied in TBI was associated with a higher median number of days until death compared to conservative treatment. A low GCS was an independent predictive factor for early intrahospital mortality in patients with TBI. In conclusion, clinical factors such as the severity of injury, low GCS, and polytrauma are predictive of early intrahospital mortality. Surgery was associated with prolonged hospitalization.

## INTRODUCTION

Traumatic brain injury (TBI) is a common cause of morbidity and mortality worldwide, with an estimated incidence of 150-300 per 100,000 people annually [1]. While some patients with TBI make a full recovery, others may suffer from permanent disabilities or die as a result of the injury. In-hospital mortality rates for patients with TBI vary widely, ranging from 11.6% to 35.3% [2]. Predicting the likelihood of death for patients with TBI is important for several reasons, including informing clinical decision-making, facilitating resource allocation, and providing prognostic information to patients and their families.

Brain edema is a common consequence of TBI and is associated with increased intracranial pressure, decreased cerebral perfusion, and poor outcomes [3]. Several studies have examined the relationship between brain edema and mortality in patients with TBI. A study by Tucker et al. found that severe brain edema was a significant predictor of in-hospital mortality in patients with severe TBI [4]. Similarly, a study by Yan et al. found that brain edema was a significant predictor of mortality in patients with TBI and hypoxia [5].

Numerous studies have identified various predictive factors for in-hospital mortality in patients with TBI. While some of these factors, such as hypoxia and biomarkers, have been extensively studied, others, such as gender, age, brain edema comorbidities, and complications, have received less attention. A study by Gao et al. found that male gender was associated with a higher mortality risk in patients with TBI [6]. However, another study by Nair et al. found that while women are overall characterized by an advantage in survival, this feature is not equally reproducible in premenopausal women [7]. The age of patients admitted due to severe TBI has increased [8]. With regard to age, a study by Fu et al. found that older age was a significant predictor of mortality in patients with TBI [9].

The severity of traumatic brain injury (TBI) is a critical predictor of patient outcomes. The Glasgow Coma Scale (GCS) is widely used as a standardized measure of TBI severity, with lower scores indicating more severe injury. Multiple studies have shown that patients with more severe TBIs, as indicated by lower GCS scores, have worse outcomes, including higher rates of mortality and morbidity. For example, a study by Rau et al. found that lower GCS scores were associated with higher mortality rates and worse functional outcomes at discharge from the hospital [10]. Another study of older patients in China found that lower GCS scores were predictive of longer hospital stays and higher rates of in-hospital mortality [11]. However, it is important to note that the predictive value of GCS may vary depending on the patient population and other factors and should be interpreted in the context of other clinical information.

Several studies have examined the relationship between comorbidities, complications, and mortality in patients with TBI. A study by Rosenfeld et al. found that the presence of comorbidities, such as hypertension and diabetes, was associated with an increased risk of mortality in patients with TBI [12]. Similarly, a study by Ho et al. found that complications, such as pneumonia and sepsis, were a significant predictor of mortality in patients with severe TBI [13].

Hemorrhage is a common complication of TBI and is associated with increased morbidity and mortality [14]. Several studies have examined the relationship between hemorrhage and mortality in patients with TBI. For example, a study by Nguyen et al. found that intracranial hemorrhage was a significant predictor of mortality in patients with TBI [15]. Similarly, a study by Wu et al. found that the severity of hemorrhage was a significant predictor of mortality in patients with TBI [16].

Identifying predictive factors for intrahospital mortality in TBI patients is essential to improving patient outcomes and guiding clinical decision-making. Understanding these predictors can help clinicians make informed decisions about patient management and improve outcomes for TBI patients. Therefore, the aim of this study was to identify the most significant predictive factors associated with the date of intrahospital death in TBI patients.

## Material and methods

The present research is a longitudinal, retrospective, analytical, observational cohort study that aimed to identify predictive factors for intrahospital death in TBI. The study population included 70 cases of TBI-related deaths admitted to the Neurosurgery Clinic from Cluj-Napoca between January 2017 and December 202.

The inclusion criteria for the study were patients who suffered TBI and died during hospitalization, while patients who died in the first 12 hours of admission were excluded from this study.

In the initial stage of the study, demographic and clinical data were recorded, including the Glasgow Coma Scale (GCS) score from the initial evaluation, TBI severity (mild, moderate, and severe), comorbidities, surgical interventions, complications that developed after admission, and the number of days of hospitalization until death occurred. Additionally, imagistic data from the initial CT scans were obtained, including primary cerebral lesions such as subdural hematoma (presence and maximal thickness), intraparenchymal hematoma (presence and maximal thickness), subarachnoid hemorrhage, diffuse axonal injuries, contusion, laceration, and cranial fracture. Secondary brain lesions were also noted, such as midline shift, brain edema, and the presence of brain herniation.

Statistical analysis was performed using the MedCalc® Statistical Software version 20.218 (MedCalc Software Ltd, Ostend, Belgium; https://www.medcalc.org; 2023). Continuous variables were tested for normality of distribution using the Shapiro-Wilk test and presented as median and 25-75 percentiles. Nominal variables were characterized by frequency and percent. Comparisons between groups were performed using Mann-Whitney or Chi-square tests whenever appropriate. Correlations between continuous variables were verified using Spearman’s rho. Variables statistically significantly associated with the number of days until death were introduced in a multivariate model. Multivariate analysis was performed using linear regression after continuous variables were log-transformed to attempt distribution normalization. A p-value <0.05 was considered statistically significant.

## Results

The association between clinical and demographic data and the number of days until death can be found in Table 1. Severe TBI was associated with fewer hospital days, as patients who died early during hospitalization did not have time to develop complications. Patients with vertebro-medullary or thoracic trauma also died more quickly. Furthermore, patients who received surgical intervention, such as craniotomy or craniectomy, had a higher median number of days until death compared to those who did not undergo surgery.

**Table 1:** Clinical and demographic data

Variables		Days until death	P-value
**Sex**	F (n=22)	4.5 (2; 10)	0.8
	M (n=48)	4.5 (2; 13.25)	
**Severity of TBI**	Mild (n=9)	11 (6.5; 32)	0.009
	Moderate (n=13)	6 (2.5; 22.5)	
	Severe (n=48)	4 (1; 7)	
**Comorbidities**	None (n=29)	4 (1; 7)	0.18
	Arterial hypertension (n=13)	4 (1.5; 15.50)	
	Chronic alcohol composition (n=9)	11 (4; 24)	
	Atrial fibrillation (n=19)	4 (2; 9)	
**Surgery**	No (n=21)	3 (1; 7.5)	0.09
	Applied (n=49)	5 (2; 15)	
**Complications**	None (n=51)	3 (1; 6)	<0.001
	Hemorrhagic shock (n=2)	7 (7; 7)	
	Bronchopneumonia (n=14)	18 (6.5; 32)	
	Septic shock (n=3)	15 (11; 0)	
**Associated lesions**	None (n=50)	5 (2.75; 14.25)	0.07
	Thoracic trauma (n=15)	2 (1; 10)	
	Thoracic and abdominal trauma (n=2)	7 (7; 7)	
	Vertebro-medullary injuries (n=3)	1 (1; 0)	

Imagistic data associated with the number of days until death can be found in Table 2. Patients without intraparenchymal hematoma or brain edema but with extended cranial fracture were more likely to die sooner.

**Table 2:** Imagistic data.

Variables		Days until death	P-value
**Brainstem injuries**	Absent (n=34)	4 (2; 14)	0.8
	Present (n=36)	5.5 (2; 9.75)	
**Diffuse axonal injury**	Absent (n=51)	6.5 (3; 15)	0.1
	Present (n=19)	4 (2; 10)	
**Subdural hematoma**	Absent (n=13)	2 (1; 19.5)	0.2
	Present (n=57)	5 (2; 10.5)	
**Intraparenchymal hematoma**	Absent (n=45)	4 (1; 8.5)	0.05
	Present (n=25)	6 (2.5; 15.5)	
**Subarachnoid hemorrhage**	Absent (n=49)	5 (2; 10)	0.7
	Present (n=21)	4 (1; 17.5)	
**Brain laceration**	Absent (n=36)	4.5 (3; 13.25)	0.4
	Present (n=34)	4.5 (1; 9.25)	
**Cranial fracture**	None (n=35)	3 (2; 8)	0.09
	Skull dome (n=17)	7 (5; 22)	
	Skull base (n=7)	6 (1; 17)	
	Skull dome and base (n=11)	2 (1; 7)	
**Brain edema**	Absent (n=13)	3 (1; 6)	0.06
	Present (n=57)	5 (2; 12.5)	
**Brain herniation**	Absent (n=44)	4.5 (1.25;14)	0.5
	Present (n=26)	4.5 (2; 7.25)	

There was a moderate positive statistically significant correlation between the number of days until death and GCS (r=0.455; p<0.001). No statistically significant correlation was observed between the number of days until death and age (r=0.080; p=0.5), subdural hematoma thickness (r=-0.040; p=0.7), intraparenchymal hematoma thickness (r=0.102; p=0.6), or midline shift (r=-0.002; p=0.9).

In order to determine which variables were independently associated with the number of days until death, a multivariate model was used (Table 3). A low GCS was associated with a shorter duration of hospitalization. Patients who died earlier did not have enough time to develop infectious complications.

**Table 3:** Multivariate analysis.

Variables	Unstandardized Coefficients		t	P-value
	B	Std. Error		
**(Constant)**	0.290	0.091	3.179	0.002
**GCS**	0.037	0.014	2.648	0.01
**Intraparenchymal hematoma**	0.074	0.101	0.730	0.4
**Complications**	0.539	0.111	4.845	<0.001

## Discussion

The results of this study may contribute to the development of a comprehensive and reliable predictive model for TBI-related deaths. By identifying these predictive factors, clinicians may improve the management and treatment of patients with TBI, potentially leading to better outcomes and reduced mortality rates. Our study demonstrated an independent significant association between the initial GCS and early intrahospital mortality in patients with TBI. Recent studies have suggested that the association between GCS and mortality in TBI patients may be related to the extent of brain damage and impaired cerebral autoregulation [17,18]. A low GCS score reflects a more severe injury to the brain, which may lead to increased intracranial pressure, reduced cerebral blood flow, cerebral edema, and brain herniation, resulting in secondary brain injury and poor clinical outcomes.

Furthermore, patients with a low GCS score are at higher risk of respiratory and cardiovascular complications, which can also contribute to early mortality [11]. Additionally, impaired cerebral autoregulation, commonly seen in severe TBI, may further exacerbate brain damage and contribute to early mortality [19]. Several potential mechanisms have been proposed to explain the relationship between GCS and mortality in TBI patients. For instance, GCS may reflect the extent of axonal injury, which is a major contributor to mortality in TBI patients [20]. Additionally, GCS may be associated with systemic inflammation and coagulopathy, which can further increase the risk of mortality [21,22].

In our study, patients who died early during hospitalization did not exhibit infectious complications. One possible mechanism is the impairment of the immune system in patients with severe TBI. Studies have shown that TBI can lead to a dysregulation of the immune system, resulting in a decrease in the production of cytokines, which are essential for the immune response. This can lead to an inability to fight off infections and may explain why patients with TBI who survive for longer periods are at a higher risk of developing infections [23]. Another potential explanation is the disruption of the blood-brain barrier in patients with TBI. The blood-brain barrier plays a crucial role in preventing the entry of pathogens into the brain [24]. However, TBI can cause damage to the blood-brain barrier, allowing bacteria and other pathogens to enter the brain and cause infections. Patients who die quickly after TBI may not have had enough time for the blood-brain barrier to be compromised, which could explain why they do not develop infections [25]. In addition, it has been suggested that the use of antibiotics and other prophylactic measures in the management of TBI may contribute to the prevention of infections in patients who die quickly after injury. These measures may be less effective in patients who survive for longer periods due to the development of resistance and other factors [26,27]. The lack of infections in patients with TBI who die quickly after an injury is a complex phenomenon that may be explained by various mechanisms, including immune dysfunction, blood-brain barrier disruption, and prophylactic measures.

Surgery was associated with longer survival in our study, although it was not statistically significant, probably due to the small sample size. Surgery is often necessary to manage severe TBI cases, such as those with large hematomas or significant mass effects. However, the decision to perform surgery on TBI patients is complex and should be made on a case-by-case basis, considering various factors, including the patient's age, the severity of the injury, and the presence of other medical conditions. Several studies have investigated the association between surgery and mortality in TBI patients. Overall, the results suggest that surgery can be beneficial in some cases, particularly those with severe injuries and significant mass effects [28-30].

Thoracic trauma was more frequent among patients who died earlier but without statistical significance. Thoracic trauma can be a significant complication in patients with TBI, and it is associated with an increased risk of mortality. Several studies have investigated the relationship between thoracic trauma and mortality in TBI patients. One study published in the Journal of Trauma found that patients with TBI and concomitant chest trauma had a higher mortality rate than those without chest trauma [31]. Another study also found that the severity of chest trauma was directly related to mortality, with patients who sustained severe chest trauma having a higher mortality rate than those with mild or moderate chest trauma [32]. The presence of rib fractures was associated with an increased risk of mortality in TBI patients [33]. The study also noted that the mortality rate increased with the number of rib fractures, with patients who sustained multiple rib fractures having a higher mortality rate than those with a single rib fracture. It is important to note that thoracic trauma can exacerbate TBI and lead to further complications, such as pulmonary contusions and pneumothorax. Therefore, prompt diagnosis and appropriate management of thoracic trauma are crucial in TBI patients to prevent further morbidity and mortality.

The limitations of our study include the small number of patients, the lack of predictive biomarkers, and the retrospective nature of the analysis, which may have introduced biases and limited the scope of the study.

## Conclusion

A low GCS was associated with early mortality in patients with TBI. Patients who died in the first days of hospitalization did not present infectious complications. Larger studies are required to validate our results.

## Data Availability

Further data is available from the corresponding author on reasonable request.
